# Water Intake, Water Balance, and the Elusive Daily Water Requirement

**DOI:** 10.3390/nu10121928

**Published:** 2018-12-05

**Authors:** Lawrence E. Armstrong, Evan C. Johnson

**Affiliations:** 1 University of Connecticut, Human Performance Laboratory and Department of Nutritional Sciences, Storrs CT 06269-1110, USA; lawrence.armstrong@uconn.edu; 2 University of Wyoming, Human Integrated Physiology Laboratory, Division of Kinesiology and Health, Laramie, WY 82071, USA

**Keywords:** water-electrolyte balance, drinking water, body water, water restriction

## Abstract

Water is essential for metabolism, substrate transport across membranes, cellular homeostasis, temperature regulation, and circulatory function. Although nutritional and physiological research teams and professional organizations have described the daily total water intakes (TWI, L/24h) and Adequate Intakes (AI) of children, women, and men, there is no widespread consensus regarding the human water requirements of different demographic groups. These requirements remain undefined because of the dynamic complexity inherent in the human water regulatory network, which involves the central nervous system and several organ systems, as well as large inter-individual differences. The present review analyzes published evidence that is relevant to these issues and presents a novel approach to assessing the daily water requirements of individuals in all sex and life-stage groups, as an alternative to AI values based on survey data. This empirical method focuses on the intensity of a specific neuroendocrine response (e.g., plasma arginine vasopressin (AVP) concentration) employed by the brain to regulate total body water volume and concentration. We consider this autonomically-controlled neuroendocrine response to be an inherent hydration biomarker and one means by which the brain maintains good health and optimal function. We also propose that this individualized method defines the elusive state of euhydration (i.e., water balance) and distinguishes it from hypohydration. Using plasma AVP concentration to analyze multiple published data sets that included both men and women, we determined that a mild neuroendocrine defense of body water commences when TWI is ˂1.8 L/24h, that 19–71% of adults in various countries consume less than this TWI each day, and consuming less than the 24-h water AI may influence the risk of dysfunctional metabolism and chronic diseases.

## 1. Introduction

Individuals with a normal P_OSM_ (e.g., 285–295 mOsm/kg) may be considered to be normally hydrated without regard to daily total water intake (TWI; [1]) or urinary biomarkers [2] because the brain actively regulates both total body water volume (within 0.5% day-to-day; [3]) and blood concentration (within a normal P_OSM_ range of 285–295 mOsm/kg; [4]) across a wide range of TWI (women, 1.3–6.1; men, 1.7–7.9 L/24h; [5,6]). Thus, an individual with suboptimal water intake may be evaluated to be euhydrated due to the defense of P_OSM_ through reduced urine production and other compensatory responses. However, there is no widespread consensus regarding a definition of euhydration. For example, the 2004 U.S. National Academy of Medicine (NAM) publication, which presented dietary reference intakes for water [6], included a lengthy review of water balance studies and water needs (i.e., using the stable isotope of water D_2_O) of children and adults (Table 1). However, this report concluded that: (a) individual water requirements can vary greatly on a day-to-day basis because of differences in physical activity, climates, and dietary contents; and (b) there is no single daily water requirement for a given person. As a result, Adequate Intake (AI) volumes for water (i.e., which are not daily water requirements) were developed from median TWI values in the NHANES III survey database [5]. The 2010 European Food Safety Authority (EFSA) panel utilized a different approach when developing dietary reference intakes [7]. Water AI values for various life-stage groups (Table 1) were derived from three factors: observed intakes of European population groups, desirable urine osmolality values, and desirable TWI volumes per unit of dietary energy (Kcal) consumed. Similar to the NAM report (above), however, the EFSA report stated that a single water intake cannot meet the needs of everyone in any population group because the individual need for water is related to caloric consumption, the concentrating-diluting capacities of the kidneys, and water losses via excretion and secretion. This report defined the minimum water requirement in general terms as the amount of water that equals water losses and prevents adverse effects of insufficient water such as dehydration.

Neither the NAM nor the EFSA document presented a method to assess the human water requirement of individuals, or (b) neuroendocrine data to support daily AI values for water. However, it is widely accepted that the brain constantly acts to preserve homeostasis via neuroendocrine responses which defend set points of body water volume and concentration [8]. In contrast to the methods used in the NAM and EFSA reports, we propose that minimal/baseline fluid-electrolyte regulatory responses by the brain signal body water balance (i.e., euhydration), and that increased neuroendocrine responses (e.g., plasma AVP levels) represent the threshold at which the brain begins to defend body water volume and concentration (i.e., hypohydration). This is important because no measurement or biomarker has previously been proposed to define a state of euhydration (i.e., often defined loosely as normal total body water or water balance). Furthermore, we propose that neuroendocrine thresholds, in conjunction with TWI measurements, can reveal the water intake requirement of individuals in a specific life-stage group when the turnover of body water (e.g., intake versus loss) is relatively constant (i.e., no large activity-induced sweat loss), an average diet is consumed, and ample water is available to support *ad libitum* drinking. Sedentary adults whose free-living daily activities include working in an air-conditioned office and consuming a typical Western diet represent an example of such a group. We propose this individualized physiological measurement of neuroendocrine responses as a methodological alternative to AI values (Table 1).

Thus, the primary purpose of the present manuscript is not to modify current AIs but rather to provide novel additional perspectives regarding 24-h TWI, euhydration, and human water requirements. We have analyzed the relationship between 24-h TWI values and their corresponding plasma AVP levels from multiple research studies, and identified a plasma AVP concentration that approximates the neuroendocrine response threshold for water regulation in free-living adults. Interestingly, this AVP threshold is exceeded when 24-h TWI is ˂1.8 L/24h. The second purpose of the present manuscript is to increase awareness of the importance of daily water intake, because a considerable percentage of individuals in industrialized countries consume less than the 24-h water AI that is recommended for their life stage. Evidence for this purpose exists in a growing body of recent epidemiological studies that report statistically significant relationships between chronic low daily water consumption and disease states or metabolic dysfunction.

Because methods and terminology vary across publications, we emphasize the following important definitions. The term *water in beverages* refers to water + water in all other fluids (e.g., juice, tea, coffee, milk). The term *total water intake* refers to water + water in beverages + food moisture (e.g., fruit, soup). Distinct from the term *dehydration* (i.e., the process of losing water), the term *hypohydration* is presently defined as a steady-state condition of reduced total body water.

## 2. Representative Research Evidence

As shown in Table 2, a variety of methods and theoretical approaches have influenced our present understanding and theories regarding human water intake, euhydration, hypohydration, and water requirements. The range of measured or calculated variables includes dietary macronutrients, 24-h TWI (defined above), biomarkers of hydration status, water volumes (i.e., consumed, metabolized, excreted, turnover), and fluid-electrolyte regulating hormones. Not all these methods (column 1, Table 2) have contributed in meaningful ways to organizational recommendations regarding the daily water intake required for good health (Table 1). For example, the NAM recommendations [6] include consideration of large non-renal water losses via sweating, during labor or physical activity. This is a primary reason why 24-h TWI recommendations from European and U.S. organizations differ by 1.1–1.3 L/24h, in specific life stage and sex categories (Table 1). Table 3 describes eight components of 24-h water balance, shown as the headings for columns 2-9. All these components interact with each other in a network that includes the central nervous system (CNS), oropharyngeal region, gastrointestinal tract, kidneys, neuroendocrine system, cardiovascular system, skin, and respiratory organs; feedback from one organ system affects all others, directly and/or indirectly. The components of 24-h water balance in Table 3 have contributed to international recommendations regarding the daily water intake required for good health (Table 1). For example, the NAM recommendations [6] assimilated all water balance components in Table 3, and the recommendations of the EFSA (2010) [7] emphasized both desirable urine osmolality values and the observed TWI of specific groups. 

A review of the hundreds of publications that contributed to our understanding of human water intake, euhydration, hypohydration, and water requirements is beyond the scope of this manuscript. However, the mean values (Table 3), measured variables, and reference citations (Table 2 and Table 3), although not exhaustive, represent the nature and types of meaningful available evidence regarding human water needs.

## 3. Why are Human Water Requirements Elusive?

**Figure 1:**
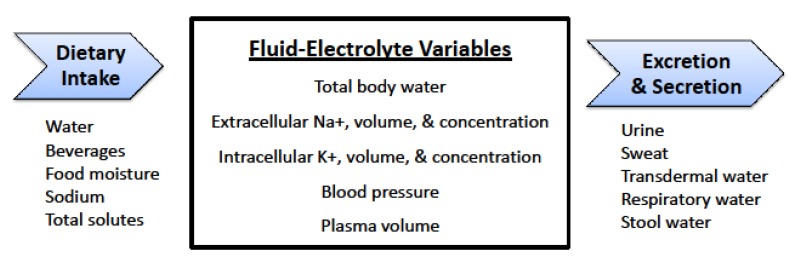
Variables that are regulated as part of body water homeostasis.

To maintain normal physiological functions (e.g.., blood pressure, pH, internal body temperature) and optimal health, and to deliver essential substances (e.g., oxygen, water, glucose, sodium, potassium) to cells, the CNS and neuroendocrine hormones act constantly to preserve internal homeostasis via a complex network of many organ and neural systems. Figure 1 presents several CNS-regulated variables which are relevant to body water balance. Each of these variables is simultaneously: (a) maintained (i.e., within the circulatory system or fluid compartments of the body) at a specific set point (e.g., a threshold beyond which the intensity of neuroendocrine responses increases ); and (b) constantly changing throughout the human life span in response to water and food intake, urine production, and non-renal water losses. Because of these fluctuations, human body water regulation is also dynamic. Therefore, we utilize the phrase dynamic complexity to refer to a constantly changing, vastly integrated regulatory mechanism [56]. This dynamic complexity is amplified by interconnected fluid compartments (i.e., intracellular, interstitial, extracellular, circulatory), organ systems (Table 3), neural plasticity (i.e., adaptations), and interactions of the physical processes (i.e., osmotic and oncotic pressure, simple diffusion, active transport) which govern water and electrolyte movements throughout the body.

**Figure 2:**
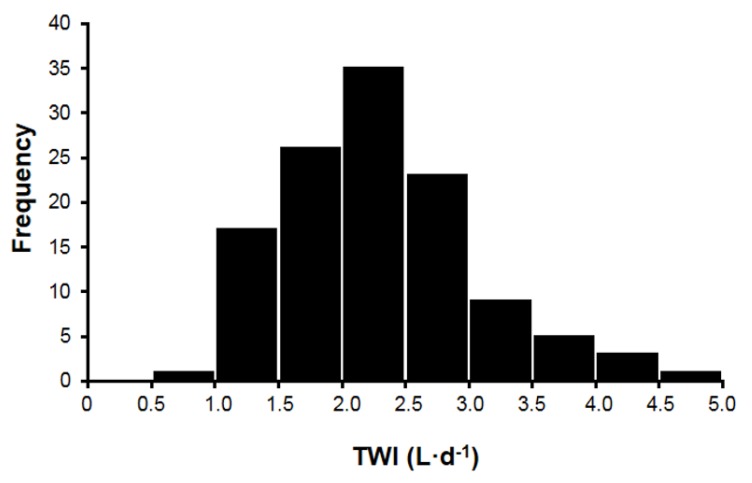
Frequency distribution of the habitual total water intake (TWI, 5-d mean values, *n* = 120) of healthy, college-aged women (*n* = 120). Reprinted with permission (Johnson et al., 2016 [13]).

This dynamic complexity (Table 3, Figure 1) represents the primary reason why the daily water requirements of humans have not been determined to this date (Table 1). We provide the following evidence in support of this statement:

- The relative influence of physiological processes which maintain water balance (Table 3) varies with different life scenarios. During sedentary daily activities in a mild environment, renal responses and thirst are the primary homeostatic regulators. During continuous-intermittent labor, or prolonged exercise at low intensities (5–18h duration), renal responses and thirst have minor-to-large effects on water regulation, whereas sweat loss presents the foremost challenge to homeostasis [56].
- Large between- and within-subject variances (i.e., of the variables in Table 3) make it difficult to determine a water requirement for all persons within a life stage (Table 1). As an example, Figure 2 illustrates the large between-subject variance of habitual TWI that exists in healthy young women (range, <1.0 to >4.5 L/24h) [13]. A large range of habitual TWI (0.6–5.2 L/24h) has also been reported for women during pregnancy [57]. Similarly, the third National Health and Nutrition Examination Survey [5] reported that the 1st decile and 10th decile of the mean TWI were 1.7–7.9 L/24h for men (*n* = 3,091) and 1.3–6.1 L/24h for women (*n* = 2,801). An example of large within-subject variability is also seen in the day-to-day differences of sweat losses that are experienced by athletes [24]. Total sweat loss during sedentary work activity (e.g., 8h of computer programming in an air-conditioned environment) may amount to <0.2 L/24h, whereas the total sweat volume during a 164-km ultradistance cycling event often exceeds 9 L during a 9-h ride [42].
- The 24-h human water requirement varies with anthropomorphic characteristics, especially body mass. Large individuals require a greater daily TWI than small individuals [6].
- The daily water requirement of any life-stage group is influenced by dietary sodium, protein and total solute load, due to individual dietary preferences as well as traditional regional-cultural foods. For example, large differences of mean urine osmolality (U_OSM_) have been reported for residents of Germany (860 mOsm/kg) and Poland (392 mOsm/kg). These differences are influenced by unique regional customs involving beverages (i.e., water, beer, wine) and food items [1] and the moisture content of solid foods; the latter factor varies among countries and demographic groups: the United States, 20–35% [2,51,58,59]; Germany, 27% [10]; the United Kingdom, 24–28%; and France, 35–38% [14].
- The principle that both water and beverages contribute to rehydration and the maintenance of body water has been fundamental in publications involving large populations [11,25], TWI differences in various countries [14,15], habitual low and high TWI consumers [16,17], water AI recommendations [6,7], the health effects of beverage consumption [60], young versus older adults [61], 12-h or 24-h water restriction [62,63], and experimental interventions which control and modify daily total water intake and beverage types [13,17,36,64]. However, small differences exist in the percentage of water retained (4-h post consumption), primarily due to beverage osmolality and the content of sodium chloride, protein, and/or energy [36,37].
- Intracellular water volume (~28 L in a 70 kg male) is considerably larger than extracellular water volume (~14 L) [65]. No hydration assessment technique measures intracellular water content or concentration directly [27].
- Although some authorities consider plasma osmolality (P_OSM_) to be the best index of euhydration and hypohydration [2,6], P_OSM_ does not assess whole-body hydration validly in all settings, especially when TBW, water intake, and water loss are fluctuating [66]. Furthermore, P_OSM_ may not reflect widely accepted physiologic principles, as shown by decreased P_OSM_ (6 out of 39 subjects) after losing 3–8% of body mass via sweating [67], and increased P_OSM_ at rest (4 out of 30 values) 60 min after ingesting 500 ml of water [68]. These findings likely result from the large between- and within-subject variance that exists in P_OSM_ measurements [56].
- Arginine vasopressin (AVP) is the body’s primary water-regulating hormone. It functions to maintain body water balance by keeping P_OSM_ within narrow limits and allowing the kidneys to alter water excretion in response to the body’s needs, in conjunction with thirst [69]. Dehydration of a large enough volume to result in increased P_OSM_ is a stimulus for the release of AVP. Table 4 summarizes research publications that determined the plasma osmotic threshold (i.e., set point) for increased plasma AVP; most of these studies employed intravenous hypertonic saline infusions with serial blood samples. Across these studies, the mean osmotic threshold values range from 280–288 and individual values range from 276–291 mOsm/kg. This large range of P_OSM_ values illustrates dynamic complexity, in that the network of fluid-regulatory functions, and water movements between fluid compartments differ across experimental designs and between normal subjects (see column 1, Table 4). Table 5 further describes the complexity of AVP, in terms of its biological functions, factors that influence neurohypohysial AVP release, and diseases which are related to AVP dysfunction.
- Thirst is the primary means by which humans sense dehydration and hypohydration. Several factors influence the onset of thirst, including blood pressure, blood volume, AVP, and angiotensin II [8]. The primary stimulus for thirst, however, is P_OSM_. Table 6 summarizes research studies which determined the plasma osmotic threshold for the appearance of thirst. Across these studies, the mean osmotic threshold values range from 286–298 and individual values range from 276–300 mOsm/kg. As with AVP (see previous item), this large range of P_OSM_ values illustrates dynamic complexity, in that the network of fluid-regulatory functions and water movements between fluid compartments differ across experimental designs and among normal subjects (Table 6). This range of P_OSM_ values also may explain part of the range in habitual TWI (Figure 2).
- Older adults (>65 years) experience reduced thirst and water intake, reduced maximal renal concentrating ability, greater plasma AVP concentration during water restriction, and reduced ability to excrete a water load when compared to younger adults [61,88,89]. Although the osmotic threshold for thirst apparently does not change during the aging process [88,90], older adults have a reduced autonomic baroreceptor capability to sense a depletion of blood volume [89,91]. In addition, older adults demonstrate changes in water satiation that hinder the ability to hydrate following an osmotic challenge. This deficiency has been linked to changes in cerebral blood flow and/or altered activation of the anterior midcingulate cortex area within the brain [92]. Thus, aging appears to be responsible for large between-subject variances (i.e., of the variables in Table 3) across age groups, which make it difficult to determine a universal water requirement for children, adults, and the elderly (Table 1).

The preceding points of evidence exemplify the difficulties which the National Academy of Medicine, USA, and the European Food Safety Authority faced and which prompted them to establish Adequate Intakes (AI), which are not Recommended Dietary Allowances (requiring a higher level of evidence) or water requirements (Table 1). The NAM assumed the TWI AI volumes to be adequate, based on observed or experimentally determined approximations or estimates of water intake by a group of apparently healthy people [6]. The EFSA determined AIs on the basis of population statistics, utilizing calculated ‘free water reserve’ (ml/24h) [12]; this quantity is defined as the difference between the measured urine volume (ml/24h) and the calculated urine volume necessary to excrete all urine solutes (i.e., obligatory urine volume, mOsm/24-h) at the group mean value of maximum U_OSM_ [1,11]. Furthermore, both the NAM and EFSA noted that AI values for water apply only to moderate environmental temperatures and moderate physical activity levels, because non-renal water losses via sweating (see column 8 in Table 3) can exceed 8.0 L/24h when exercise-heat stress is extreme [7].

## 4. A Proposed Method to Assess Daily Water Requirements

**Figure 3:**
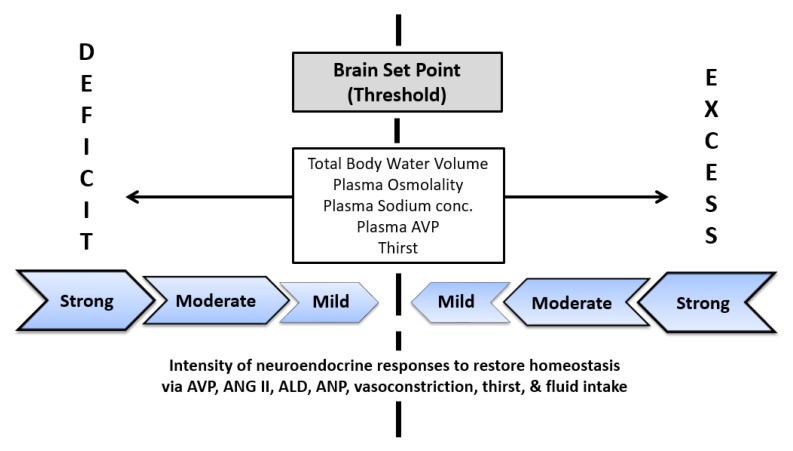
A proposed schematic of a method to assess human daily water requirements by measuring the intensity of neuroendocrine responses that are employed by the brain to defend homeostasis of body water volume and concentration. These responses and thresholds are inherent hydration biomarkers, and the means by which the brain maintains good health and optimal function. Abbreviations: AVP, arginine vasopressin; ANG II, angiotensin II; ALD, aldosterone; ANP, atrial naturietic peptide.

We now propose a novel approach to the assessment of the daily water requirement of individuals in all life stages, which was not employed during the development of water AI values (Table 1). This method focuses on the thresholds and intensity of responses within the brain and neuroendocrine system (i.e., autonomic nerves and endocrine organs that release hormones to regulate water and electrolyte balance). Figure 3 provides a graphic representation of this technique. The central dashed line represents the set point (threshold) for each of the five regulated variables listed within the central rectangle; Table 4 and Table 6 present set point values for a plasma AVP increase and the appearance of thirst. The regions to the left and right of the set point represent a water or sodium deficit, and water or sodium excess, respectively; the zones farthest to the left and right of the set point represent the greatest perturbations of each regulated variable due to change forces (e.g., dehydration, drinking, large dietary osmotic load). The block arrows to the left and right of the set point illustrate neuroendocrine responses which move each regulated variable toward the set point in an effort to restore altered homeostasis; examples include release of AVP, angiotension II, aldosterone, atrial natriuretic peptide, as well as blood vessel constriction or dilation, increased thirst, and water consumption (i.e., sensory and behavioral effects that are influenced by endocrine responses). The strongest neuroendocrine responses occur at the far left and far right of the threshold (labeled with the words deficit and excess). If all fluid-electrolyte regulatory variables are at or near the set point (or if all neuroendocrine responses are minimal), a state of euhydration exists because the brain is activating no compensatory responses; in contrast, when responses counteract water loss a state of dehydration or hypohydration exists. Measuring the intensity of neuroendocrine responses and identifying when set points have been exceeded allow for quantitative comparisons of values during controlled laboratory experiments. Alternatively, the area under the curve (i.e., response intensity plotted versus response duration) could be measured.

**Figure 4:**
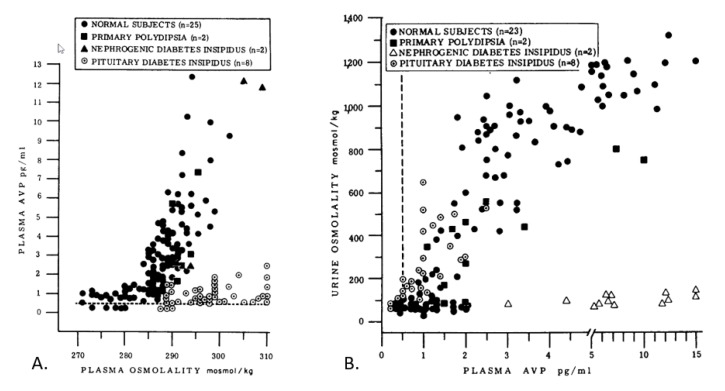
The relationship of plasma osmolality to plasma AVP (panel A), and the relationship of plasma AVP to urine osmolality (panel B). Reprinted with copyright from Robertson et al. [71]. Plasma was collected during recumbent rest in three states of water balance: ad libitum fluid intake, following an acute water load (20 ml/kg), and after acute periods of fluid restriction. The data represent healthy adults and patients with diverse types of polyuria (i.e., abnormally large urine volume and frequency). Dashed lines represent the sensitivity limit of the plasma AVP assay.

Figure 4 illustrates this approach to assessing individual daily water needs. In normal subjects, the increase in plasma AVP (panel A) is stimulated primarily by increased P_OSM_. Increasing P_OSM_ signifies increasing perturbation of homeostasis; whereas an increasing plasma AVP concentration represents an increased intensity of neuroendocrine response and indicates that the brain is regulating body water via the kidneys. Thus, the data in the upper right quadrants of panel A and panel B correspond to intense neuroendocrine responses and a rigorous defense of total body water; the data in the lower left quadrants correspond to minimal-to-moderate defense of total body water.

In normal adults, an increased intensity of neuroendocrine response (i.e., which defends the volume and concentration of total body water) results in decreased urine volume and increased urine osmolality, secondary to increased plasma AVP (Figure 4, panel B). As such, urinary variables (e.g., osmolality, specific gravity, 24-h urine volume) have been identified as valid hydration biomarkers in studies involving free-living pregnant women, nonpregnant women, and men [16,18,38,39,57]. Central, autonomically-controlled changes of plasma AVP concentration (i.e., at the border of euhydration and mild hypohydration; Figure 3) also act to maintain optimal health and functions in normal persons. In turn, AVP may be a prognostic indicator of various disease states (Table 7), including ischemic stroke, myocardial infarction, pneumonia, certain types of cancer, and septic shock [93,94,95].

**Figure 5:**
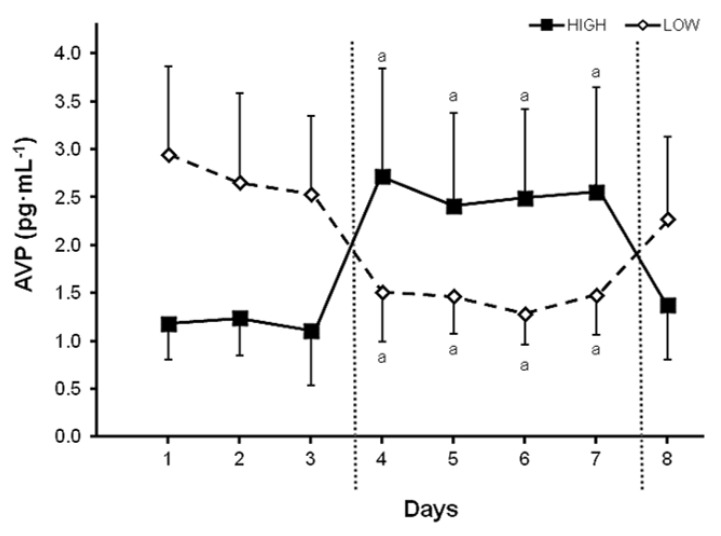
Morning plasma AVP concentrations of habitual high-volume drinkers (HIGH, 3.2 ± 0.6 L/24h, *n* = 14♀) and low-volume drinkers (LOW, 1.6 L/24h, *n* = 14♀) during ad libitum baseline (3 days), modified water intake (4 days; HIGH, 2.0 ± 0.2 and LOW, 3.5 ± 0.1 L/24h), and ad libitum recovery (1 day; HIGH, 3.2 ± 0.9 and LOW, 1.7 ± 0.5 L/24h). Different experimental phases are separated by vertical dotted lines. a, within-group significant difference from the 3-d baseline mean (*p* < 0.001). Reprinted with permission from Johnson et al., (2016) [13].

In terms of the water requirements of normal individuals, determining the intensity of the body’s defense of total body water and tonicity (e.g., measuring changes of plasma AVP or regulated variables) provides a laboratory method to assess the intensity of homeostatic responses and the response thresholds which the brain employs. Once identified, these measurements could be compared to experimentally-controlled TWI volumes to determine the minimum 24-h TWI that generally elicits no neuroendocrine response above resting baseline levels (i.e., thereby representing euhydration or normal water balance). This method for assessing 24-h water balance also can be applied to the TWI of free-living adults. For example, in recent years several research teams have compared the physiological responses of habitual low-volume drinkers (LOW) to those of habitual high-volume drinkers (HIGH) [16,17,18,51]. Figure 5 depicts plasma the AVP concentrations of free-living LOW and HIGH during one morning laboratory visit on each of 8 days. The TWI levels of LOW (*n* = 14 ♀) and HIGH (*n* = 14 ♀) are described in the figure legend. The experimental design involved 3 d of baseline observations, 4 d of modified water intake (during which LOW consumed the TWI which HIGH habitually consumed, and vice versa), and 1 d of *ad libitum* water intake. The morning plasma AVP levels in Figure 5 were similar (LOW, 1.4–1.5 pg/ml; HIGH, 1.1–1.3 pg/ml) when both groups were consuming a similar high TWI (LOW, 3.5 L/24h; HIGH, 3.2 L/24h). Considering the plasma AVP threshold which indicates an obvious neuroendocrine response (~2.0 pg/ml [78]; Figure 4), LOW were above this threshold when consuming a TWI volume of 1.6–1.7 L/24h whereas HIGH were above this 2.0 pg/ml AVP threshold only when their TWI was modified to 2.0 L/24h on days 4–7. We interpret these data to mean that the brain did not attempt to conserve water when TWI was ≥3.2 L/24h, and that the water requirement of these healthy young women existed between 1.6 and 3.2 L/24h. Similar plasma AVP concentrations have been published in a study of LOW (TWI, 0.74 L/24h) and HIGH (TWI, 2.70 L/24h) by Perrier and colleagues [16]. Furthermore, a plasma AVP threshold ˂2.0 pg/ml corresponds closely with the euhydrated baseline values shown in Table 7 (1.0–1.8 pg/ml), as observed in four groups of men and women. However, when these test subjects underwent water restriction for 12h and 24h, a stronger neuroendocrine response was observed (i.e., representing hypohydration) as plasma AVP levels of 2.9–3.5 pg/ml in young adults and 8.3 pg/ml in older adults. In one Table 7 experiment [63], participants (5 ♂ & 3 ♀, 26–50 years) rehydrated with tap water (10 ml/kg, 620–870 ml) after a 24-h water restriction; 60 min after this water consumption, the average plasma AVP decreased from 3.3 to 1.5 pg/ml, suggesting that subjects had reached a state of euhydration.

Utilizing a plasma AVP threshold of ˂2.0 pg/ml, this method could be employed to: (a) define and distinguish states of euhydration and hypohydration; and (b) evaluate the neuroendocrine response changes which occur with advanced age (Table 7; [61,88,91,96]) or any factor that potentiates the release of AVP into the circulation. Although a plasma AVP concentration is useful because it represents the sum of all factors that influence pituitary AVP release (Table 5) and AVP turnover in the circulation, we acknowledge that other hormonal/neurological biomarkers (e.g., angiotensin II, aldosterone) also play a role in water homeostasis. In addition, cases of over-hydration likely represent a limitation of this method. Excess body water may reduce plasma AVP to a level below the sensitivity of present-day technologies (i.e., most immunoassays detect AVP to ≥0.5 pg/ml; [69], making it difficult to distinguish over-hydrated states from a normal euhydrated state (1.0–1.8 pg/ml; Table 7). One final limitation of this method is the circadian variation in AVP secretion. It would be imperative for any investigation utilizing this proposed method to ensure blood sampling at similar times if successive samples were taken as it has been demonstrated that AVP is both an outcome and input related to suprachiasmatic nucleus activity [97].

Plasma AVP concentration was selected as the primary outcome variable of this method because previous TWI investigations have also published AVP with no copeptin measurements. However, AVP has a short half-life and is difficult to isolate/analyze [69,98], whereas copeptin is stable at room temperature and is recognized as a diagnostic biomarker for various diseases [99,100]. Furthermore, several studies have reported a strong correlation between plasma AVP and copeptin levels in healthy individuals [98,101] and patients [102,103] across a wide range of P_OSM_. Thus, it is very likely that copeptin will be measured in future investigations of neuroendocrine response intensity. 

Currently, copeptin is just beginning to be used in clinical settings during randomized control trials evaluating changes in water intake and disease [104,105]. Clinical settings are ideal because copeptin measurement, although less prone to errors compared to AVP, still requires specialized and expensive equipment (i.e., B·R·A·H·M·S KRYPTOR random-access immunoassay analyzer, ThermoFisher Scientific). Copeptin is also utilized in hospital settings for the evaluation of heart failure [106]. Therefore, many researchers in the clinical setting already have access to copeptin analysis equipment. It is hoped that in the coming years this type of equipment will become a mainstay within hydratrion physiology laboratories, or advances in assay techniques will make copeptin quantification more accessible for all levels of researchers.

To our knowledge, only one study has assessed changes of plasma copeptin concentrations in normal adults during water restriction [107]. Resting euhydrated plasma copeptin values (i.e., representing no neuroendocrine response by the brain to conserve water) were 18.5 ± 6.8 pg/ml (4.6 ± 1.7 pmol/L; 8 ♀, 8 ♂) at baseline, and increased to 37.0 ± 20.9 pg/ml (9.2 ± 5.2 pmol/L) after a 28-h water restriction period that induced a 1.7% body mass loss. To determine a plasma copeptin threshold similar to the plasma AVP threshold of 2.0 pg/ml (see above), additional experiments similar to those in Table 7 are required.

## 5. Neuroendocrine Responses across a Range of TWIs

**Figure 6:**
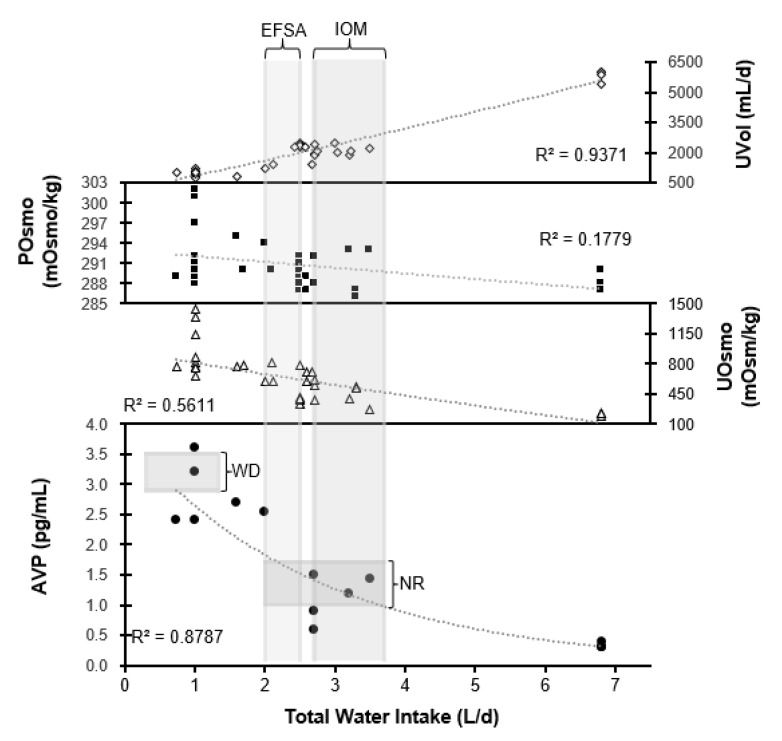
Urine volume (U_VOL_), plasma osmolality (P_OSM_), urine osmolality (U_OSM_, 24h), and plasma AVP plotted against daily total water intake. EFSA and NAM water Adequate Intakes are shown as the vertical shaded column. AVP concentrations associated with water restriction (WR) and baseline resting total water intake (B) (Table 7) appear as horizontal shaded rows. All data points are group mean values (SD not shown) from investigations that measured TWI.

Figure 6 provides evidence regarding the daily water requirement of humans. Compiling a range of data from six studies that reported 22 different observation days [10,13,16,18,54,64], we plotted the relationships between TWI and the primary brain-regulated variable (P_OSM_), the neuroendocrine response (plasma AVP) to changes of P_OSM_, and the resulting changes in urine volume and concentration. The R^2^ value for each relationship describes the amount of variance in the four variables that is explained by TWI values. Clearly, P_OSM_ is not strongly related to TWI (*R*^2^ = 0.18, *p* < 0.05) because blood concentration is regulated by the brain within a narrow normal limit [4], across a wide range of TWI [6]; as reported in previous publications [16,18], P_OSM_ does not serve as a valid indicator of either TWI or water requirement in free-living adults. This fact opposes the theory that all individuals with normal P_OSM_ levels are similar [2,108], even if their 24-h TWI is low.

The relationship between TWI and urine osmolality in Figure 6 appears to be moderately strong (*R*^2^ = 0.56, *p* < 0.001), but not very strong because of the simultaneous influence of dietary osmolar load on U_OSM_. In contrast, the relationship between TWI and urine volume is very strong (*R*^2^ = 0.94, *p* < 0.001). However, plasma AVP concentration is the only variable in Figure 6 that directly represents the intensity of neuroendocrine responses across a wide range of TWI (0.7–6.8 L/24h). As such, this relationship (*R*^2^ = 0.88) provides evidence of the TWI volume that is required to maintain water and electrolyte homeostasis. Recalling that a plasma AVP concentration of ≥2.0 pg/ml indicates an obvious neuroendocrine response by the brain ([78]; Figure 4), and that the normal resting level (shaded zone labeled WR) of plasma AVP lies below 2.0 pg/ml, the line of best fit for the relationship between TWI and plasma AVP in Figure 6 shows that a plasma AVP concentration of 2.0 pg/ml is equivalent to a TWI of 1.8 L/24h. Approximately 40% of young healthy college-aged women in the USA (Figure 2), 19–24% of men and women in Great Britain [109], and 68–71% of men and women in Spain [110] consume less than this volume of water each day. In Figure 6, a TWI of 1.8 L/24h is equivalent to a urine volume of ~1400 mL/24h and a 24-h urine osmolality of ~770 mOsm/kg. Furthermore, a TWI of 1.8 L/24h is similar to the much-debated recommendation to drink “8 × 8” each day, which refers to consuming eight 8 oz glasses of water (1.89 L/24h) [108,111], and to the recommended daily AI of water for women (2.0 L/24h, Table 1) established by the European Food Safety Authority [7]. The two vertical shaded zones in Figure 6 display the EFSA and NAM daily water AI ranges for men and women; all of these AI correspond to plasma AVP levels <2.0 pg/ml and a minimal/baseline neuroendocrine defense of total body water and tonicity (Table 7). The daily water AI range of the NAM corresponds to plasma AVP levels well below 2.0 pg/ml, likely because the NAM proposed that human water requirements should not be based on a “minimal” intake [6], as this might eventually lead to a deficit and possible adverse performance and health consequences [2].

Figure 6 also depicts plasma AVP levels during the four 12-h and 24-h water restriction experiments (see the horizontal shaded zone labeled WR) that are described in Table 7. At the end of these water restriction periods, group mean plasma AVP concentrations ranged from 2.9–3.5 pg/ml, representing a mild-to-moderate neuroendocrine response (Figure 3 and Figure 4). Interestingly, this range of concentrations (representing hypohydration of approximately 1% of body mass) is similar to the plasma AVP levels reported for individuals who habitually consume a low daily TWI of 0.7 L/24h (2.4 pg/ml; [16]), 1.0 L/24h (2.5–3.6 pg/ml; [54]), and 1.6 L/24h (2.5–2.9 pg/ml; Figure 5; [13]). This suggests that women who consume a TWI of 0.7–1.6 L/24h (i.e., ~20–30% of young healthy women; Figure 2)—well below the AI recommended by EFSA and NAM (Table 1)—experience a chronic mild-to-moderate neuroendocrine defense of total body water. This observation is significant because numerous investigations and letters to the editor have proposed that chronically elevated plasma AVP and angiotensin II levels may be related to negative health outcomes (e.g., cardiovascular disease, obesity, diabetes, cancer morbidity and mortality) [69,96,112] as well as the progression of disease states (e.g., salt-sensitive hypertension, chronic kidney disease, and diabetic nephropathy [113]). Other investigators have: (a) utilized plasma copeptin concentrations (i.e., part of the molecular pre-prohormone of AVP) to detect myocardial ischemia and other diseases (Figure 4; [8,69,114]); and (b) supported the use of AVP receptor antagonists to treat specific cardiovascular pathologies, suggesting dysfunctional body water regulation [115,116]. A 6-week study, involving 82 healthy adults (50% ♀) in three TWI groups (1.43, 1.83, and 2.42 L/24h), provided promising evidence (i.e., increasing the daily TWI of the two LOW groups to match 2.42 L/24h) of reduced circulating copeptin levels [19]. This study suggests that the reduction of plasma AVP via increased TWI offers a safe, cost-effective, and easy-to-implement primary preventive intervention that should be evaluated in large future clinical trials [117].

## 6. Evidence for a Role of 24-h TWI in Reducing Disease Risk

As noted above, a surprising number of adults in developed countries do not meet water AI recommendations (Table 1). This fact is significant in terms of long-term health outcomes because a growing body of epidemiological evidence shows that chronically elevated plasma AVP ( likely due to an insufficient daily TWI) is related to cardiovascular, obesity and cancer morbidity and mortality, as well as the regulation of glucose metabolism. Unfortunately, controlled, randomized clinical trials (spanning multiple years or decades), which focus on the relationship between chronic low water volume intake and development of diseases, do not exist for three reasons [118]. First, lengthy controlled studies suffer from participant attrition and noncompliance because it is difficult for any person to maintain a constant hydration state or 24-h TWI across years of life. Second, multiple personal characteristics, dietary habits, or lifestyle behaviors may concurrently encourage disease development. Third, because of the large number of confounding dietary, behavioral, and genetic factors related to the development of the above diseases, the number of subjects required for adequately-powered statistical analyses which isolate the effects of water is large and the research is costly. Therefore, researchers must focus on the mechanisms that regulate body water (Figure 1 and Figure 4) to investigate potential relationships between chronic low TWI and the development of diseases.

Recent epidemiological studies (*n* = 3000–4000 Scandinavian adults) have reported statistically significant associations between high plasma AVP (or its surrogates, including low daily TWI, low urine flow rate, high P_OSM_, or copeptin) [69] and the incidence of type 2 diabetes, metabolic syndrome, end-stage renal disease, cardiovascular disease, and premature death [117,119,120,121,122]. Furthermore, during the past 25 years, several observational studies have reported that increased daily TWI reduces the risk of kidney stone formation and stone recurrence [118,123,124,125,126,127,128] preserves kidney function in chronic kidney disease [129], and retards cyst growth in polycystic kidney disease [130]. If these findings are supported by future controlled intervention studies, it is possible that increased daily water consumption will be recognized as a safe, cost-effective, simple primary preventive intervention for some kidney diseases [117,131]. However, establishing such relationships will be challenging (i.e., for the reasons noted in the preceding paragraph) [118], and will likely be specific to each type of kidney disease/dysfunction. For example, at least one recent randomized clinical trial found no benefit of coaching chronic kidney disease patients to increase their 24-h TWI, even though coaching resulted in a 24-h urine volume that was +0.6 L/day greater than an uncoached control group (*p* < 0.001) [104]. Thus, because epidemiological and observational studies do not allow for cause-and-effect inferences, randomized clinical trials and experimental studies employing animal models are needed to further evaluate mechanisms such as the contribution of AVP to renal, cardiovascular, and metabolic disorders [113]. 

## 7. AVP Influences Glucose Metabolism

The first observations regarding the association between “pituitary extract” and antidiuresis occurred more than a century ago [132], after which AVP synthesis and its mechanistic description became the subjects of the 1955 Nobel Prize in Chemistry [133]. Two decades later, medical professionals hypothesized that the excess water loss (i.e., polyuria) observed in people with type 2 diabetes mellitus resulted from decreased secretion of AVP. Contrary to their inclination, researchers testing this hypothesis observed that the plasma AVP concentration was markedly *elevated* in hyperglycemic patients [134]. These researchers, along with many during subsequent decades, hypothesized that increased plasma AVP was an ineffective effort to conserve water in the face of an overwhelming solute diuresis caused by glucose in urine (i.e., glucosuria). To our knowledge, this theory persisted until 2010, when epidemiological findings reversed the relationship between AVP and glucose regulation, from one of result to cause [135]. Based on an apparent dose-response relationship between copeptin and severity of the metabolic syndrome [136], the important work of Enhörning and colleagues [135] described a significant positive association between the baseline plasma copeptin concentration of healthy adults and their odds of developing impaired fasting glucose and/or diabetes during the five-year observation period. Shortly thereafter, it was confirmed that an inverse relationship existed between self-reported water intake and the development of hyperglycemia, during a nine-year longitudinal surveillance study [137]. These two investigations were theoretically connected by the fact that AVP is secreted in response to high plasma osmolality (i.e., low water intake) and acts via V2 receptors in renal nephrons, increasing translocation of the aquaporin 2 channel and facilitating increased water reabsorption [138]. Thus, chronically low water intake is associated with elevated plasma AVP [16], both of which predict the development of impaired glucose regulation.

Historically, low daily water intake, in the absence of symptoms and outside of athletic pursuits, has been considered to be innocuous because theoretically it was balanced by increased AVP secretion, enhanced renal water reabsorption, increased water consumption, and maintenance of water balance [108]. However, the examination of cellular receptors sensitive to AVP yielded insights into possible mechanisms responsible for the above results. Distinct from V2 membrane receptors, V1a receptors are expressed in the kidney, liver, vascular smooth muscle, blood platelets, and brain [69]. In the liver, V1a stimulation by AVP initiates calcium signaling reactions which increase glycogenolysis and blood glucose, directly in hepatocytes and indirectly via vasoconstriction-mediated ischemia [139]. Indeed, multiple animal investigations and human genetic studies have described the role of V1a receptors in glucose regulation and dysfunction [140,141,142].

Independently, V1b receptors are concentrated within the hypothalamic-pituitary-adrenocortical (HPA) axis, where they enhance ACTH and cortisol release, and the physiological effects of CRH, when stimulated by AVP. The downstream effect of increased plasma glucocorticoids is increased hepatic gluconeogenesis and decreased insulin sensitivity [143]. The role of AVP in glucocorticoid release is supported by observational data which show that individuals who consume low daily TWI exhibit increased plasma concentrations of both AVP and cortisol [16]. Additionally, men with type 2 diabetes exhibited deteriorated glucose control and increased plasma cortisol following three days of water restriction [144]. Thus, mounting evidence suggests that a link exists between AVP and glucose regulation. Our understanding of this relationship will expand in coming years.

## 8. Summary

Despite numerous efforts to define a state of euhydration and determine the daily water requirements of children, men, women, and older adults, no empirical research provides definitive answers and no universal consensus exists. The dynamic complexity of the water regulatory network, and inter-individual differences, are the primary reasons why widespread consensus regarding the daily water requirements has not been reached to this date. In the preceding paragraphs, we have proposed a novel experimental approach that offers an alternative to water AI survey data and the potential to determine 24-h water requirements of individuals. This approach involves the assessment of the intensity of neuroendocrine responses during euhydration and following experimental perturbations of essential regulated variables. Although future researchers may choose a different regulated variable, we focused on plasma AVP as the primary hydration biomarker to determine the intensity of neuroendocrine responses to a range of TWIs (Figure 6). A plasma AVP concentration < 2.0 pg/ml represents a baseline euhydrated state (i.e., the brain is not attempting to conserve water), whereas plasma AVP ≥2.0 pg/ml indicates dehydration or hypohydration because the brain is acting to conserve water ([78]; Figure 4). These concentrations provide a previously unpublished definition of each term. Our examination of data from multiple research studies (Figure 6) demonstrates that a plasma AVP concentration of 2.0 pg/ml is equivalent to a TWI of 1.8 L/24h. Observational studies demonstrate that 19–71% of adults in various countries consume this volume of water or less each day [13,109,110]. This is significant because increasing evidence shows that chronically elevated plasma AVP (likely due to an insufficient daily TWI) could contribute to a number of negative health outcomes. A TWI of 1.8 L/24h also questions the theory that all individuals with normal P_OSM_ levels are similar [2,108], even if their 24-h TWI is low. Fortunately, the chronic reduction of plasma AVP by consuming a daily TWI that maintains plasma AVP <2.0 pg/ml suggests a safe, cost-effective, and easy-to-implement primary preventive intervention that can be evaluated in future long-term clinical trials.

## Figures and Tables

**Table 1:** Comparison of recommended Adequate Intakes ^a^ for water, published by European and American health organizations.

Life Stage & Sex	Age	European Food Safety Authority, Parma, Italy ^b^ 2010 (ml/day)	National Academy of Medicine, USA 2004 ^b^ (ml/day)
Infants	0–6 months	680 via milk	700
	6–12 months	800–1,000	800
Children	1–2 years	1100-1200	1300
	2–3 years	1300	
	4–8 years	1600	1700
	9–13 years, boys	2100	2400
	9–13 years, girls	1900	2100
	14–18 years, boys	2500	3300
	14–18 years, girls	2000	2300
Adults			
Men		2500	3700
Women		2000	2700
Pregnant Women	≥ 19 year	2300	3000
Lactating Women	≥ 19 year	2600–2700	3800
Elderly		same as adults	same as adults

^a^ Adequate Intakes represent an amount that should meet the needs of almost everyone in a specific life-stage group who is healthy, consumes an average diet, and performs moderate levels of physical activity [6,7]; ^b^, values refer to total water intake (TWI = plain water + beverages + food moisture).

**Table 2:** Investigational and theoretical approaches to assess human water intake, euhydration, hypohydration, and water requirements.

Methods	Variables Measured or Calculated	Relevance		Critique	Representative Publications
		Individuals	Group		
Partitioning 24-h urine production into minimum urine volume ^a^ and free urine volume ^b^	U_VOL,_ U_MAX,_ U_VM,_ U_FUV_	X		U_MAX_ is determined via observations of a few males and was applied to individuals. U_MAX_ varies with age and had a large inter-subject variability.	[9]
Calculation of free water reserve ^c^ to determine individual 24-hour hydration status ^d^	U_VOL,_ U_MAX,_ U_VM,_ U_FWR,_ U_OSM_, U_TOT,_ NRWL		X	This population-based method updates the concepts of Gamble (above), does not determine the U_MAX_ of individuals, and estimates NRWL. In single (<24h) samples, confounding factors ^e^ may dominate and other hydration biomarkers are preferred.	[10,11,12]
Dietary recall to determine TWI	plain water, beverages, food moisture	X	X	Data are specific to the subject sample, and typically do not provide information regarding water balance or turnover.	[13,14,15]
Responses and hydration biomarkers of free-living LD ^f^ versus HD ^f^	U_OSM_, U_SG_, U_COL_, U_VOL_, P_OSM_, S_OSM_, M_B_		X	Studies assess the responses of adult groups who have habitually different TWIs.	[13,16,17,18,19,20]
Global, regional, and country water consumption recommendations	TWI (L/24 h)		X	Adequate intakes ^g^ for TWI are based on survey data median values.	[6,7,21]
Statistical categories of hydration status for free-living adults	U_OSM_,U_SG_, U_COL_, U_VOL_, P_OSM_, M_B_	X		Seven categories range from euhydrated to hypohydrated or hyperhydrated. Variables are expressed per single sample and 24-h collection.	[22,23]
Laboratory water turnover and movement, using the DLW technique or stable isotope of water ^h^	^2^H_2_^18^O, ^2^H_2_O, TBW, U_VOL_, NRWL	X		Mean water turnover (L/24h) incorporates estimates of TWI, metabolic, transcutaneous, and inspired air water.	[22,24,25]
Water balance of free-living adults during daily activities	TBW, TWI, U_OSM_, U_VOL_, P_OSM_, TPP, HCT, SR, M_B_	X	X	Various methods are used to describe the water needs of specific life stage and sex groups.	[2,25,26]
Laboratory controlled experiments evaluating dehydration and rehydration	U_OSM_,U_SG_, U_COL_, U_VOL_, P_OSM_, S_OSM_, M_B,%_∆PV	X		Dehydration is accomplished via passive exposure to a hot environment, exercise, or water restriction. Rehydration is accomplished via water and beverage intake or intravenous fluid administration.	[27,28,29,30,31,32]
Laboratory investigations that focus on thirst sensations and drinking behavior	TWI, beverages, U_OSM_, U_VOL_, P_OSM_,%∆PV, AVP	X			[33,34,35]
Laboratory comparison of beverages: rehydration efficacy	FC, U_VOL_, BHI	X		Common beverages are evaluated to identify retention (relative to still water) in euhydrated, but not dehydrated, adults. The diuretic response is influenced by fluid characteristics including osmolality, energy density, and electrolyte content.	[36,37]
Plasma AVP or copeptin ^i^ responses	AVP, copeptin ^i^	X	X	The hormone AVP maintains U_VOL_, P_OSM_, and body water balance within narrow limits, in conjunction with thirst.	[13,16,19,31]
Assessment of specific urine and plasma hydration biomarkers	U_OSM,_ U_COL_, P_OSM,_ P_OSM_:U_OSM_ ratio	X	X	Most studies focus on the assessment of simple, practical hydration biomarkers for use during daily activities.	[16,18,38,39,40,41]
Field studies of hydration status during labor, exercise, or competition	TWI, SR, P_OSM_, U_OSM_, U_SG_, U_COL_, M_B_	X		Research attempts to optimize health and performance.	[42,43,44,45]
Statistical and graphical determination of the probability of dehydration	P_OSM_, U_SG_, M_B_	X	X	Predictions are based on a modest dehydration range (−2.1 to −3.5% M_b_) in 6 men and 5 women.	[46]
Calculated biological variation and diagnostic accuracy of dehydration biomarkers	P_OSM_, S_OSM_, U_OSM_, U_SG_, U_COL_, M_B_	X	X	Statistics evaluate biomarkers, on the basis of a functionally important range of −2.0 to −7.0% M_b_, induced in 5 women and 13 men across x hours.	[47]
Theoretical consideration of intracellular and extracellular dehydration	P_Na+_, P_OSM_, S_OSM_, U_OSM_, U_SG_, U_COL_, M_B_	X		Candidate biomarkers of dehydration must consider intracellular, extracellular, and mixed dehydration stimuli.	[48]

^a^, minimum urine volume corresponds to the urine volume necessary to excrete urine solutes at maximum urine osmolality (defined as 1400 mOsm/kg); ^b^, free urine volume is a precursor to the modern concept of free water reserve (see Table 1); ^c^, free water reserve is calculated statistically as the virtual water volume that could be additionally reabsorbed at maximum osmolality, in all but 2% to 3% of healthy subjects at a specific life stage and sex; ^d^, collection of a 24-h urine sample, determination of urine volume and osmolality, and calculation of obligatory and free water volumes allow for the determination of individual 24-h hydration status, determined using statistical confidence intervals (Manz and Wentz, 2005 [11]); ^e^, e.g., meal timing and contents, physical activity; ^f^, LD and HD were defined slightly differently in each study (LD range, 1.0–1.6; HD range, 2.4–3.3 L/24h); ^g^, adequate water intake is not a requirement, but rather the TWI that meets the needs of almost everyone in a specific life stage and sex group, to prevent deleterious effects of dehydration (i.e., metabolic and functional abnormalities); ^h^, the DLW method is theoretically based on the differential turnover kinetics of the stable isotopes of oxygen (^18^O) and hydrogen (^2^H). After drinking a known mass of DLW (^2^H_2_^18^O), ^2^H is eliminated from body water as H_2_O whereas ^18^O is eliminated as H_2_O and CO_2_ (Racette et al., 1994 [49]). The accumulation of a stable isotope of water (^2^H_2_O) in plasma, saliva, urine, or sweat determines the rate of water movement throughout the body. ^i^, AVP is difficult to measure because of its brief half-life, whereas plasma copeptin is relatively stable and its concentration is strongly correlated to that of AVP. Abbreviations: AI, adequate intake; AVP, arginine vasopressin; DLW, doubly labeled water; HCT, hematocrit; HD, individuals who habitually consume a high daily water volume; LD, individuals who habitually consume a low daily water volume; M_B_, body mass; NRWL, non-renal water loss as eccrine sweat, transdermal, respiratory and stool water; S_OSM_, salivary osmolality; SR, sweat rate measured as M_B_ change; TBW, total body water; TWI, total water intake = (plain water + water in beverages + food moisture); T_OSM_, tear osmolality; FC, fluid consumed during a defined time period; BHI, beverage hydration index, relative to water;%∆PV, percent change of plasma volume; TPP, total plasma protein; U_COL_, urine color [50]; U_FUV_, free urine volume; U_FWR_, free water reserve; U_OSM_, urine osmolality; U_TOT_, total excreted osmolar load; U_SG_, urine specific gravity; U_VM_, minimal urine volume; U_VOL_, urine volume; U_MAX,_ maximal urine osmolality produced by the kidneys;.

**Table 3:** Dietary, physiological, metabolic, and behavioral components of human 24-h water balance.

	Total water intake ^a^ (L/24h)	Intracellular metabolic water production ^b^	Total solute load ^c^ (mOsm/24h)	Urine osmolality ^d^ (mOsm/kg)	Maximal renal concentrating ability (mOsm/kg)	Urine volume (L/24h)	Non-renal water loss (L/24h) ^e^	Free water reserve ^f^ (L/24h)
Functions and characteristics	Contributes to TBW	Product of human metabolism	Metabolized and digested products excreted in urine	Regulates TBW and ECV-ICV osmolality	Inherent quality of the kidneys	Regulates TBW and ECV-ICV osmolality	Excretory and secretory processes	Calculated index of euhydration, based on population statistics
Influential factors	Meal timing and contents, idiosyncratic thirst, physical activity, body size, cultural and learned preferences	Metabolic rate and substrates, physical activity, diet macronutrient and energy content, NES responses	Metabolic products, dietary contents, body size, idiosyncratic hunger, learned food preferences	TWI, MRCA, solute load, NRWL, physical activity, NES responses	Life-stage group, male or female sex	TWI, total solute load, NRWL, physical activity, NES responses	Diet, ventilatory rate, physical activity, body size	TWI, total solute load, NRWL, physical activity
Organs involved	GI, CNS, NES, mouth and throat	CNS, NES	GI, CNS, NES	Kidneys, CNS, NES	Kidneys, CNS, NES	GI, kidneys, CNS, NES	Skin, GI, respiratory organs	GI, kidneys, CNS, NES
Conscious or behavioral influence?	Yes, habitual 24-h water intake	No	Yes, solid food consumption	Yes, secondary to TWI and food contents	No	Yes, secondary to TWI and food contents	Yes, eccrine sweat loss during labor or exercise	Yes, secondary to water and food intake
Representative mean, median, or range of values for sedentary adults	♀, 1.8–2.0 and ♂, 1.9–2.4 (FR, UK); ♀&♂, 1.5–2.5 (13countries); ♀, 2.3 (range: 0.8–4.5) (USA); ♂, 3.0 (range: 1.4–7.7) and ♀, 2.5 (range: 1.2–4.6) (USA); ♀, 1.9 and ♂, 2.3 (GE); ♀&♂, 0.2–3.9 (FR) L/24h	♀, 0.2–0.3; ♂, 0.3–0.4; ♂, 0.4 L/24h	♀, 669–781 and ♂, 915–992 (GE); ♂, 951 (USA); ♀&♂, 362–1365 (4 countries); ♂, 750 (USA); ♀, 752 and ♂, 941 (GE) mOsm/24h	♀&♂, 120–1250 (FR); ♀&♂, 555 (UK) mOsm/kg	♀&♂, 1430 (UK) mOsm/kg; ♀&♂ range, 1100–1300 (GE); ♀&♂, 1010–1330 (USA)	♀&♂, 0.2–3.9 (FR); ♀&♂, 1.9 (*n* = 8, UK) L/24h	♂, 0.3–0.4 (UK); ♀, 0.5–0.7 and ♂, 0.7–1.3 (GE) L/24h	♀, 0.4–0.5 and ♂, 0.2–0.3 (GE); ♀, 0.5 and ♂, 0.3 (GE) L/24h
Reference citations	[11,14,15,25,38,51]	[25,52,53]	[1,11,12,41]	[38,54]	[1,54,55]	[38,54]	[1,24]	[11]

^a^, TWI, total water intake = (plain water + water in beverages + food moisture); ^b^, water generated during substrate oxidation; ^c^, greatly influenced by diet composition; ^d^, in a 24-h sample; ^e^, NRWL includes eccrine sweat, transdermal, respiratory and stool water losses; ^f^, FWR = (24-h urine volume, L/day) − (obligatory urine volume, L/day). The latter term is the water volume necessary to excrete the 24-h solute load, hypothetically calculated as (830 mOsm/kg) − (3–4 mOsm/kg per year > 20 years of age) [1]. Hydration status is inadequate if FWR is negative. Abbreviations: TBW, total body water; NES, neuroendocrine system (central nervous system + hormones); ECV, extracellular volume; ICV, intracellular volume; MRCA, maximal renal concentrating ability; CNS, central nervous system (brain + spinal cord); GI, gastrointestinal organs; GE, Germany; FR, France; USA, United States of America.

**Table 4:** Plasma osmotic threshold ^a^ for plasma AVP increase.

Osmotic Threshold ^b^ (mOsm/kg)	Participants/Conditions	References
282 (280–285)	Normal adults (*n* = 6♂), dehydration via water restriction, upright posture	Moses and Miller, 1971 [70]
285 (284–286) ^c^	Normal adults (*n* = 9♂), IV_HS_	Moses and Miller, 1971 [70]
287 (286–288) ^c^	Normal adults (*n* = 6♂), IV_HS_	Moses and Miller, 1971 [70]
288 (287–289) ^c^	Normal adults (*n* = 6♂), IV_HS_, then IV_HS_ plus dextran (expanded plasma volume)	Moses and Miller, 1971 [70]
280 (272–284) ^c^	Normal adults (*n* = 25), recumbent rest, in three states: *ad libitum* fluid intake, acute water load (20 ml/kg) and water restriction	Robertson et al., 1973 [71]
280 (276–291) ^c^	Normal adults (*n* = 9♂, 7♀), recumbent rest	Robertson et al., 1976 [72]
IV_HS_, 287 (283–291) M, 286 (282–290)	Normal adults (3♀, 3♂), supine rest, IV_HS_ (5%) and hypertonic mannitol (M, 20%)	Zerbe et al., 1983 [73]
285 (282–289)	Healthy adults (10♂), recumbent rest, IV_HS_ and IV_I_	Thompson et al., 1986 [74]
287 (286–288)	Healthy adults (7♂), recumbent rest, IV_HS_	Thompson et al., 1988 [75]
287 (281–290)	Healthy adults (3♂, 4♀), recumbent rest, IV_HS_	Thompson et al., 1991 [76]
MZ, 283 (277–290) DZ, 281 (274–285)	Healthy twins (7♂ monozygotic pairs, 6♂ dizygotic pairs), IV_HS_	Zerbe et al., 1991 [77]

^a^, refers to the plasma osmolality (i.e., determined statistically or graphically) at which plasma AVP concentration rises from baseline; ^b^, mean (range or 95% confidence interval); ^c^, data derived from a figure.

**Table 5:** Research findings that illustrate the dynamic complexity of AVP, a peptide hormone produced in the hypothalamus ^a^.

**Biological functions**
Regulates body water and sodium homeostasis by acting on renal nephrons to decrease urine volume and increase the concentration of urine Maintains plasma osmolality within narrow limits Encourages vasodilation in vascular smooth muscle cells by inducing NO production Affects liver metabolism (e.g., gluconeogenesis, glycogenolysis) Stimulates the pancreas to produce either insulin or glucagon, depending on blood glucose concentration
**Factors which influence neurohypohysial AVP release and plasma AVP concentration**
Plasma osmolality Angiotensin II Oropharyngeal environment Water restriction and consumption Osmolar content of diet, especially sodium Hypoglycemia Blood volume and pressure Upright posture Emotional stress Exercise Circadian rhythmicity Hypoxia Nausea Pain
**Diseases and disorders that involve AVP dysfunction**
Diabetes insipidus and diabetes mellitus Syndrome of inappropriate ADH excess (SIADH) Sepsis Severe bleeding, hemorrhage Chronic hypernatremia Primary polydipsia syndrome, compulsive water drinking Kallmann’s syndrome Autosomal dominant polycystic kidney disease

^a^, compiled from: [69,72,78,79,80,81,82,83,84,85,86].

**Table 6:** Plasma osmotic threshold ^a^ for appearance of the thirst sensation.

Mean (Range ^b^) Osmotic Threshold (mOsm/kg)	Participants/Conditions	References
IV_HS_, 298 (294–300) M, 296 (290–299)	Normal adults (*n* = 2–5 ♂&♀), supine rest, IV_HS_ (5%) and hypertonic mannitol (M, 20%)	Zerbe et al., 1983 [73]
F, 297 (296–298) L, 293 (291–295)	Healthy women (*n* = 8) were tested in the follicular (F) and luteal (L) phases of the menstrual cycle, IV_HS_	Spruce et al., 1985 [87]
287 (286–288)	Healthy males (*n* = 7), recumbent rest, IV_HS_	Thompson et al., 1988 [75]
287 (282–291)	Healthy adults (3♂, 4♀), recumbent rest, IV_HS_	Thompson et al., 1991 [76]
MZ, 286 (276–293) DZ, 289 (283–296)	Healthy twins (7♂ monozygotic pairs, 6♂ dizygotic pairs), IV_HS_	Zerbe et al., 1991 [77]

^a^, refers to the plasma osmolality (i.e., determined statistically or graphically) at which thirst is first perceived; ^b^, mean (range or 95% confidence interval); Abbreviations: IV_HS_, intravenous hypertonic saline; IV_I_, intravenous isotonic saline.

**Table 7:** Effects of 12-h and 24-h water restriction ^a^ on plasma osmolality and AVP concentration.

Participants	Experimental Design Phase	Plasma Osmolality (mOsm/kg H_2_O)	Plasma AVP (pg/ml)	Reference
8 ♀ ^b^ (21–34 year)	Baseline, EU	289 ± 2	1.3 ± 0.6	Davison et al., AJP 1984 [62]
	12-h WR ^c^	294 ± 2	2.9 ± 1.2	
5 ♂ & 3 ♀ ^b^ (26–50 year)	Baseline, EU	292 ± 1	1.7 ± 0.2	Geelen et al., AJP 1984 [63]
	24-h WR ^c^	302 ± 1	3.3 ± 0.5	
7 ♂ (20–31 year)	Baseline, EU	288 ± 1	1.0 ± 0.3	Phillips et al., NEJM 1984 [88]
	24-h WR ^c,d^	291 ± 1	3.5 ± 0.3	
7 ♂ (67–75 year)	Baseline, EU	288 ± 1	1.8 ± 0.3	Phillips et al., NEJM 1984 [88]
	24-h WR ^c,d^	296 ± 1	8.3 ± 0.3	

^a^, diets included no water or beverages and dry food items; ^b^, nonpregnant women; ^c^, 24-h total water intake was not measured; ^d^, body mass loss was 1.8–1.9% of the baseline value; Abbreviations: AVP, arginine vasopressin; EU, euhydrated; WR, water restriction.
